# An evolving landscape: predictive and therapeutic biomarkers in advanced non-small cell lung cancer

**DOI:** 10.3389/fonc.2026.1750153

**Published:** 2026-03-11

**Authors:** Dylan Fortman, Angel Qin

**Affiliations:** Department of Medicine, Division of Hematology and Oncology, University of Michigan, Ann Arbor, MI, United States

**Keywords:** Biomarker, emerging, immunotherapy, NSCLC, targeted therapy

## Abstract

Lung cancer remains the leading cause of cancer-related mortality. The incorporation of immune-checkpoint inhibitors and targeted therapies have revolutionized the treatment landscape in non-small cell lung cancer (NSCLC). Comprehensive testing for actionable biomarkers, including immunohistochemistry, fluorescence *in situ* hybridization, and next generation sequencing is critical for patients with NSCLC to ensure accurate therapeutic management. In this review, we summarize the methodology and molecular testing for oncogenic driver alterations in NSCLC, highlight current biomarkers in advanced NSCLC predictive of response to immunotherapy and targeted therapies with a focus on recent drug approvals, and discuss future directions in this rapidly advancing field.

## Introduction

Lung cancer is the second most commonly diagnosed malignancy in the U.S. with over 200,000 estimated new cases in 2025 and is the leading cause of cancer-related death (1). Non-small cell lung cancer (NSCLC) accounts for approximately 87% of new lung cancer diagnoses, with a majority of new diagnoses being advanced-stage disease (2, 3). Survival trends have improved over the last few decades with expanded therapy options. However, five-year relative survival across all stages of NSCLC remains poor and is estimated to be 27.9%, with stage IIIA, IIIB, and IV disease having an estimated five-year overall survival (OS) of 26.2%, 17.3%, and 5.8%, respectively (2, 4). For patients who have advanced-stage without curative options, such as surgical resection or definitive chemoradiotherapy, and for patients with metastatic disease, the mainstay of therapy remains palliative systemic treatment.

Systemic treatments have evolved over the last few decades with the advent of immunotherapy (checkpoint inhibitors) and targeted therapies for specific oncogenic driver alterations. For patients with locally advanced/metastatic disease not amenable to curative therapy and whose tumors do not harbor targetable alterations, combination chemoimmunotherapy is the standard of care first-line therapy, while single-agent immunotherapy (IO) is an option for select patients (5–11). Oncogenic alterations can be identified in approximately 60% of lung adenocarcinoma. Certain oncogenic alterations, such as *EGFR*, occur more frequently in females, individuals with minimal or no prior tobacco exposure, and patients of East Asian descent; in contrast, other alterations, including *KRAS* and *BRAF*, are observed across both sexes and in individuals regardless of smoking history (12, 13). Alterations in epidermal growth factor receptor (*EGFR*) and anaplastic lymphoma kinase (*ALK*) have been well known to confer resistance to standard immunotherapy-based regimens, with multiple retrospective studies showing lower objective response rate (ORR) and progression-free survival (PFS) across a range of molecular subgroups (14–16); a notable exception includes *Kirsten rat sarcoma virus* (*KRAS*) mutations which appear more commonly in patients with smoking history and which do not appear to alter response to immunotherapy-based regimens (17, 18). The NCCN guidelines thus recommend tumor testing for oncogenic driver mutations for all patients with advanced nonsquamous NSCLC and NSCLC not otherwise specified (NOS) to help guide systemic treatment decisions. Molecular testing should also be considered in select patients with squamous histology as well (19).

**Figure 1:**
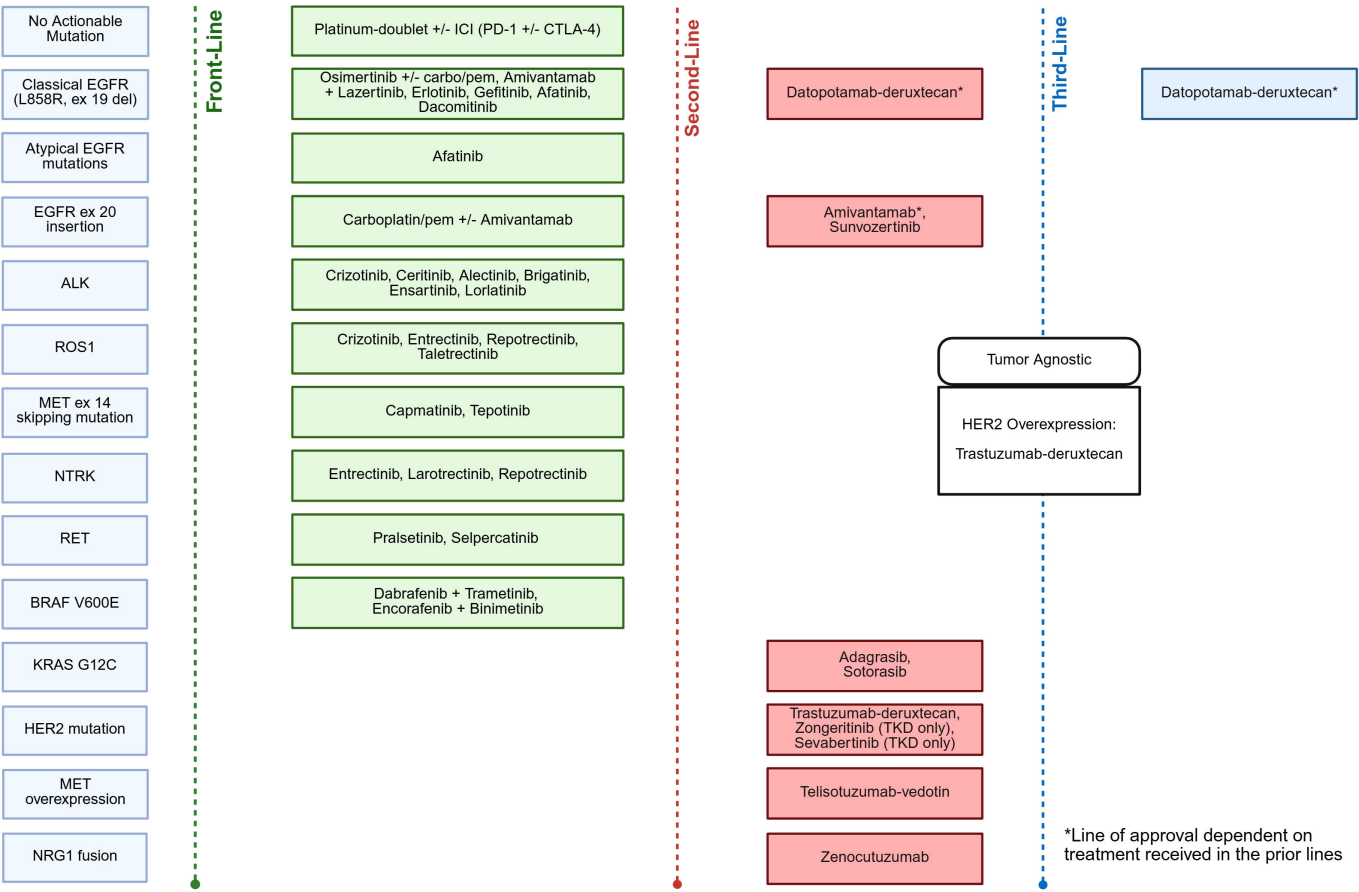
Current targeted therapy approvals in advanced/metastatic NSCLC.

Over the past decade, there has been an increasing number of actionable biomarkers predictive of response to immunotherapy and targeted therapies. With the incorporation of immunotherapy and targeted therapies in early-stage NSCLC, along with a plethora of approved agents in the front-line metastatic setting (**Figure 1**), the need for comprehensive molecular testing is paramount. In this review, we aim to review the methodology and molecular testing for oncogenic driver alterations in NSCLC, highlight current biomarkers in advanced NSCLC predictive of response to immunotherapy and targeted therapies with a focus on recent drug approvals, and discuss future directions in this rapidly advancing field.

## Overview of molecular methodology

Broadly speaking, current methods of identifying actionable oncogenic alterations (AGAs) in NSCLC include immunohistochemistry (IHC), fluorescence *in situ* hybridization (FISH), and next generation sequencing (NGS). IHC utilizes antigen-antibody complexes against specific cell-surface proteins to evaluate protein expression. IHC remains the sole method for detecting PD-L1 expression, which is required to calculate the TPS, a key metric in NSCLC treatment decision-making; specifically, a minimum of 100 viable tumor cells are assessed for PD-L1 expression, with the TPS expressed as a percentage by dividing the number of PD-L1 positive tumor cells with the total number of viable tumor cells and multiplying by 100. Additionally, IHC can detect MET overexpression, human epidermal growth factor receptor 2 (*HER2, ERBB2*) overexpression, and specific DNA rearrangements or point mutations, such as B-Raf proto-oncogene (*BRAF*) V600E point mutation, *ALK*, neurotrophic tropomyosin receptor kinase (*NTRK*), and proto-oncogene tyrosine-protein kinase-1 (*ROS1*) translocations (20, 21). FISH utilizes single-stranded DNA or RNA probes which can bind to their molecular sequencing counterparts and therefore allow visualization (fluorescence) of any present alteration. FISH is largely considered gold standard for the detection of gene amplifications (*MET* and *ERBB2*); it can additionally be used for DNA rearrangement confirmatory testing following IHC (*ALK, NTRK, ROS1*, and rearranged during transfection gene (*RET*)), however, this can be costly and requires adequate tissue sample (20, 22).

Genomic profiling using NGS may involve analysis of a broad, cancer-relevant gene panel applicable to all solid tumor types, a more targeted panel specific to a particular tumor type, or specific and focused single-gene testing. NGS can identify the main classes of genomic alterations, including insertions/deletions, base substitutions, amplifications and homozygous deletions, and gene fusions/rearrangements; moreover, microsatellite instability status (MSI) and tumor mutation burden (TMB) can be identified (23). Comprehensive genomic profiling (CGP), if available, is preferred compared to single-gene testing. Tissue samples are gold standard for NGS testing, however, liquid biopsies are available for isolating circulating tumor DNA (ctDNA) if tissue sample is limited (20).

Importantly, commercial NGS platforms vary in their capabilities, and physicians should recognize limitations related to cost, tissue requirements, and the depth and breadth of biomarker coverage. Notably, RNA-based NGS identifies approximately 15-20% more patients with actionable structural variants compared to DNA-NGS alone (24) Furthermore, RNA-based NGS overcomes fundamental limitations of DNA-based fusion detection by directly observing expressed fusion transcripts and splicing junctions rather than attempting to sequence through large, repetitive intronic regions (24); one example is the detection of Neuregulin 1 (NRG1) fusions, as the gene spans only 1.4 megabases and only 0.3% of the gene encodes the protein (25).

Although NGS testing is often regarded as a comprehensive approach for identifying genetic alterations beyond PD-L1 TPS, reliance on this method alone in this current era of targeted therapies may overlook drug approvals based off IHC-detected protein overexpression, such as MET and HER2 overexpression (26–28). Therefore, NCCN and other consensus guidelines endorse the use of a broad, multi-modality approach. Nonetheless, despite the growing adoption of molecular testing practices and NCCN recommendations to perform such in all advanced non-squamous NSCLC and NSCLC-NOS cases, real-world data indicate that this remains underutilized, particularly in community settings and among patients facing socioeconomic barriers (29–31). Given the increasing number of actionable mutations in NSCLC, the ability to have comprehensive biomarker testing is critical for patients to receive appropriate standard of care therapies.

Lastly, while not validated nor currently recommended for widespread use, the incorporation of artificial intelligence (AI)-derived biomarkers for predicting response to systemic therapies is an emerging platform. Recently, the trophoblast cell surface antigen 2 (TROP-2) normalized membrane ratio, measured through AI-based quantitative continuous scoring, has shown to be the first AI-derived predictive biomarker of response to targeted therapy with the antibody–drug conjugate (ADC) datopotamab deruxtecan (32). This development is particularly significant as it addresses a broader challenge in ADC development: the need for predictive biomarkers beyond simple target expression; the application of AI to create more sophisticated, quantitative assessments of target proteins represents an important step forward in personalizing ADC therapy and improving patient selection.

## Biomarkers of immunotherapy response

### Overview

Programmed death-1 (PD-1) is a glycoprotein expressed on the surface of activated immune cells while its ligands – PD-L1 (B7-H1) and PD-L2 (B7-DC) – have distinct expression patterns, with PD-L1 constitutively expressed at low levels on antigen-presenting cells (APCs) as well as other nonhematopoietic cell types, whereas PD-L2 is expressed on dendritic cells and macrophages (33). Under physiologic conditions, the PD-1/PD-L1 pathway prevents excessive T-cell activity which could lead to autoimmunity; in the tumor microenvironment, upregulation of PD-L1 and its binding to PD-1 effectively leads to immune escape via decreased T-lymphocyte activation and proliferation along with T-cell apoptosis and modulated activity of CD4/8+ T-cells, NK cells, and macrophages (33–36). Cytotoxic T-lymphocyte associated protein 4 (CTLA-4) is expressed solely on T-cells and acts as a decoy receptor for the downregulation of immune response due to its higher affinity for B7 than the T-cell co-stimulatory protein CD28 (37, 38). Immune checkpoint inhibitors (ICI), such as PD-1/L1 and CTLA-4 inhibitors, thus allow for immunologic reconstitution and the ability for cytotoxic T-cell destruction of tumor cells.

Immunotherapy has revolutionized the treatment of multiple types of malignancies, including non-AGA NSCLC. In early stage, resectable NSCLC, the use of nivolumab, pembrolizumab, or durvalumab in combination with chemotherapy as neoadjuvant and/or perioperative therapy prior to surgical resection has become the standard of care (39–43). Similarly, adjuvant pembrolizumab or atezolizumab are approved for select patients after surgical resection for early stage NSCLC on the basis of PD-L1 TPS, while consolidation durvalumab after definitive concurrent chemoradiotherapy for unresectable stage III NSCLC is approved regardless of PD-L1 TPS (44–46). In the advanced or metastatic setting, combination chemoimmunotherapy (chemoIO) is the standard of care frontline therapy, with single-agent immunotherapy (IO) remaining an option for select patients (5–11). In this section, we will summarize biomarkers predictive of response to immunotherapy, with a focus on PD-L1 TPS, tumor mutational burden (TMB), and tumor-infiltrating lymphocytes (TIL); additionally, we will discuss emerging biomarkers and negative predictors of response to immunotherapy.

### PD-L1 TPS

To date, PD-L1 expression remains the best available biomarker guiding immunotherapy-based treatment selection in advanced or metastatic NSCLC. Single-agent PD-1/PD-L1 inhibitors have shown improved tolerability and clinical outcomes compared to chemotherapy in the front-line setting for patients with PD-L1 TPS ≥ 50% (11, 47, 48). In one study, patients with very high PD-L1 expression, defined as TPS ≥ 90%, had improved ORR (60.0% versus 32.7%), longer median PFS (14.5 vs 4.1 months), and longer median OS (not reached vs 15.9 months) compared to patients with PD-L1 TPS 50-89% when treated with first-line pembrolizumab, suggesting higher PD-L1 TPS is predictive of more favorable response to ICI (49). For patients with PD-L1 TPS < 1% or between 1 – 49%, first-line combination chemoIO is standard of care as previously mentioned; notably, pembrolizumab has FDA approval for PD-L1 positive disease, defined as TPS >1%, albeit the OS benefit appears exclusive to patients with PD-L1 TPS ≥ 50% (50). Combination ICI therapies are approved in the front-line setting, including nivolumab-ipilimumab with or without chemotherapy, along with durvalumab-tremelimumab with chemotherapy, with the former demonstrating improved outcomes regardless of PD-L1 TPS (including TPS < 1%) and the latter having superior benefit for PD-L1 TPS ≥ 50% (51–53).

Across histologies, PD-L1 expression appears to function differently in squamous cell NSCLC than in non-squamous NSCLC; in a retrospective, multicenter analysis, PD-L1 expression levels (TPS < 1%, 1-49%, ≥ 50%) in non-squamous NSCLC correlated with significant stepwise improvement in PFS and OS while the benefit in squamous cell is dichotomized (<1% vs ≥ 1%) (54). Moreover, responses to ICI therapy in squamous cell carcinoma are less consistent than those observed in non-squamous histologies, as combinations of atezolizumab or durvalumab-tremelimumab with chemotherapy have not demonstrated an OS benefit in patients with squamous cell histology compared to other ICI-based regimens (53, 55). Novel prospective clinical trials are needed in this space.

Despite the reliance of PD-L1 TPS in therapeutic ICI-selection, it remains an imperfect biomarker. Among patients with a PD-L1 TPS of ≥ 50%, durable disease control is observed in approximately 20% of patients, while about 20% exhibit early disease progression and roughly 60% experience delayed progression following initial disease stabilization (56). Moreover, multiple studies have demonstrated benefit with the use of ICI therapy irrespective of PD-L1 expression levels (57, 58). PD-L1 expression is dynamic and heterogenous within tumors, introducing potential inaccuracies related to tumor sampling across different anatomical sites (primary vs metastatic lesions) and timepoints (59). Lastly, variability in IHC platforms and positivity thresholds further highlights a lack of standardization for PD-L1 TPS (60). Nevertheless, PD-L1 remains the most established biomarker for immunotherapy response in NSCLC.

### TMB and TIL

Tumor mutational burden (TMB), defined as the total number of mutations per megabyte of exonic regions of evaluated genes in a tumor specimen (mut/Mb), within tumors leads to the production of immunogenic antigens and activation of T-lymphocyte-induced immune response (61, 62). Studies have shown that higher tumor mutational burden is associated with increased response rates to PD-1–based therapy across multiple tumor types (63–66). Pembrolizumab received tumor agnostic-approval in 2020 for the treatment of advanced or metastatic tumors with high TMB, defined as ≥ 10 mut/Mb, based on results from the phase II KEYNOTE-158 basket trial and remains the only ICI agent approved based on TMB alone (67); notably none of the patients in this trial had NSCLC. Preliminary data from CheckMate 227 with ipilimumab and nivolumab in metastatic NSCLC had suggested TMB might be predictive of response to ICI therapy; however, updated data demonstrated OS was improved regardless of TMB (51, 68). Given these data, and concern on the lack of standardized measurement and cutoff for designating TMB “high” disease, the NCCN does not recommend routinely checking TMB when determining treatment decision with immunotherapy in NSCLC.

Tumor infiltrating lymphocytes (TIL) serve as both prognostic and predictive biomarkers of immunotherapy response in NSCLC. The prognostic value of TILs has been supported by retrospective studies in NSCLC and other malignancies, with higher TIL density associated with improved overall survival (69–71). In a metanalysis of over 15,000 patients with NSCLC, elevated TILs correlated with favorable prognosis, particularly among CD8+ T-cell subtypes (72). The predictive significance of TILs has been supported in a real-world retrospective cohort of patients with advanced NSCLC treated with nivolumab, in which a high TIL burden (≥10%) was associated with a median PFS of 13 months vs 2.2 months in low-TIL patients (73). For patients with NSCLC who received anti-PD-1 therapy, one study utilizing a machine-learning-based TIL scoring approach demonstrated combined models of TILs/PD-L1 or TMB/PD-L1 had improved specificity in determining response to ICI therapy compared with PD-L1 alone; interestingly, in the PD-L1 TPS < 1% subgroup, TIL levels had superior accuracy for determining ICI-response compared to TMB (74). Standardization efforts are ongoing to establish reproducible quantification methods to complement existing biomarkers for immunotherapy response, including PD-L1 TPS and TMB.

### Emerging biomarkers of response to immunotherapy

There is a plethora of emerging biomarkers of positive and negative responses to immunotherapy in NSCLC. Genomic alterations are ongoing area of exploration. It is well known that *EGFR* and *ALK* alterations portend poor response to ICI therapy, while *KRAS* mutations do not appear to alter response (14–18). Mutations in serine/threonine kinase 11 (*STK11*) and Kelch-like ECH-associated protein 1 (*KEAP1*), in isolation or concurrently with *KRAS* mutations, have been shown to predict primary resistance to ICI therapy while mutations in *TP53* correlate with improved response rates and survival in advanced NSCLC treated with ICI (75, 76). Notably, the combination of CTLA4-inhibitors with PD-(L)1 blockade appears to have the capability to overcome to PD-(L)1 inhibition alone in patients with STK11 and/or KEAP1 mutations (76). In a retrospective analysis of the POSEIDON trial, patients with *STK11*, *KEAP1*, and *KRAS* mutations had improved OS with addition of tremelimumab to durvalumab and chemotherapy backbone compared to chemotherapy and durvalumab alone, suggesting patients with these mutations may benefit from a triplet regimen (76, 77); however, more prospective studies are needed for patients with *STK11* and/or *KEAP1* mutations to identify the optimal systemic therapy strategy. The ongoing phase III TRITON study evaluating tremelimumab and durvalumab versus pembrolizumab, in combination with chemotherapy, in non-squamous advanced or metastatic NSCLC with *STK11*, *KEAP1*, and/or *KRAS* mutations will help address this question (NCT06008093).

Aside from genetic alterations, the tumor microenvironment (TME) has become a hotbed of active research, including analysis of tertiary lymphoid structures (TLS) and multiplex immunohistochemistry for detecting the spatial relation of intratumoral protein co-expression patterns; with respect to predictors of immune checkpoint inhibitor efficacy, both the density and maturation status of TLS in NSCLC appear to be associated with improved clinical outcomes while multiplex IHC demonstrates superior positive prediction of ICI response compared to PD-L1 alone (60, 78). Other areas of ongoing investigation include peripheral circulating biomarkers (ctDNA – circulating tumor DNA) and the microbiome.

## Biomarkers with approved targeted therapies

### EGFR

In lung adenocarcinomas, epidermal growth factor receptor (*EGFR*) mutations are identified in approximately 40–60% of Asian patients and in 20–30% of Caucasian patients, with a higher prevalence among female non-smokers or light smokers; the majority of these mutations involve exons 18–21 (79, 80). The most common *EGFR* mutations, termed “sensitizing mutations” due to their susceptibility to EGFR tyrosine kinase inhibitors (TKIs), are exon 19 deletions (Ex19del, 45%) and the exon 21 *L858R* point mutation (40%) (81). Less common sensitizing mutations, including *G719X*, *S768I*, and *L861Q*, exhibit variable responsiveness to EGFR TKIs and are typically referred to as atypical mutations. In contrast, exon 20 insertions (Ex20ins) confer primary resistance to first- and second-generation TKIs, with low response to third-generation TKIs (82–84).

In advanced NSCLC with common sensitizing EGFR Ex19del or *L858R* alterations, there are several approved first-line agents, including erlotinib (with or without either ramucirumab or bevacizumab), gefitinib, dacomitinib, osimertinib (with or without platinum-based chemotherapy), and amivantamab-lazertinib combination therapy (85–92). First-line osimertinib monotherapy was approved on the basis of the phase III FLAURA trial (90); more recently, the phase III FLAURA2 trial demonstrated improved outcomes with combination osimertinib and platinum-based chemotherapy (91, 93). To address common resistance mechanisms such as *MET* amplification, the phase III MARIPOSA trial evaluated the bispecific EGFR–MET antibody amivantamab in combination with the EGFR TKI lazertinib as first-line therapy, compared with osimertinib or lazertinib monotherapy, and demonstrated improved outcomes (94, 95). Additional studies of combination regimens are ongoing in the front-line setting, such as the phase II RAMOSE clinical trial evaluating combination osimertinib and bevacizumab compared to osimertinib monotherapy in metastatic TKI-naïve *EGFR-*mutated disease (96).

In the second-line setting for patients who progress on osimertinib monotherapy, several treatment options exist, including amivantamab in combination with platinum-based chemotherapy, adding platinum-based chemotherapy to continued osimertinib backbone therapy, and datopotamab deruxtecan (Dato-DXd), a trophoblast cell surface antigen 2 (TROP-2) antibody drug conjugate (ADC) linked to topoisomerase-I microtubule inhibitor payload (97–100). Additional studies investigating the use of Dato-DXd are ongoing, including the TROPION-Lung14 phase III clinical (NCT06350097) and the TROPION-Lung15 phase III clinical trial (NCT06417814). Additional TROP-2-targeted ADCs are being developed, with sacituzumab tirumotecan showing promising activity in this space (101). Finally, other ongoing areas of research include targeting specific resistance mechanisms (phase III SAFFRON trial; NCT05261399) and evaluating the role of novel, bispecific PD-1/VEGF inhibitors (phase III HARMONi trial; NCT06396065).

Studies for patients with uncommon or “atypical” sensitizing EGFR mutations (*G719X*, *S768I*, *L861Q*) are limited to non-randomized trials with variable efficacy among agents. Recent evidence suggests that subgrouping EGFR mutations using a structure–function–based approach provides enhanced insight into drug sensitivity predictions; for example, second-generation EGFR-TKIs such as afatinib appear be more selective than third-generation TKIs in P-loop αC-helix compression (PACC) mutants (*G719X, S768I, L747P/S, V769L*, etc) (102). Currently, only afatinib has FDA approval for patients with atypical EGFR mutations.

Patients with Ex20ins tend to have poorer outcomes compared to their counterparts, owing to altered conformation at the receptor kinase-active site, limiting TKI binding and rendering first-, second-, and third-generation EGFR TKI largely ineffective (82–84). Historically, platinum-based chemotherapy had been the mainstay of treatment until the phase III PAPILLON trial led to the approval of combination amivantamab with platinum-based chemotherapy (103). More recently, sunvozertinib, a selective, irreversible EGFR TKI has been granted accelerated FDA approval in July 2025 for use in second-line Ex20ins advanced disease (104). Sunvozertinib monotherapy is being investigated in the front-line setting in the ongoing WU-KONG-15 trial (NCT05559645) with early data showing promising results (105). Zipalertinib, an oral irreversible EGFR TKI, and furmonertinib, a CNS-potent broadly-active EGFR-TKI, have shown promising activity in the phase I/II open-label REZILIENT1 trial (NCT04036682) and phase Ib FAVOUR trial (NCT04858958), respectively (106–108); these agents are both being explored in the front-line setting for advanced NSCLC with EGFR Ex20ins (NCT05973773, NCT05607550). Lastly, enozertinib, a highly brain-penetrant, irreversible EGFR inhibitor with activity against atypical EGFR mutations and exon 20 insertions has demonstrated promising phase I clinical data, with an ongoing study assessing the combination of enozertinib with subcutaneous amivantamab in advanced or metastatic NSCLC with exon 20 insertions (NCT06816992) (109).

Given the benefit of EGFR TKIs demonstrated in advanced disease, the incorporation of EGFR TKIs in early-stage NSCLC with EGFR sensitizing mutations has also been investigated. In the phase III ADAURA trial, three years of adjuvant osimertinib resulted in a 12% improvement in 5-year OS (110). Similarly, the phase III LAURA trial demonstrated improved PFS with the use of osimertinib vs placebo in unresectable stage III NSCLC after definitive chemoradiotherapy in patients with Ex19del or L858R (111). More recently, neoadjuvant osimertinib has been studied in the NeoADAURA trial with improved rates of pathologic complete response compared to neoadjuvant chemotherapy (112). In an era of improved OS and pathologic outcomes with neoadjuvant or perioperative chemoimmunotherapy in EGFR-WT resected NSCLC, these data with earlier incorporation of targeted therapy is encouraging.

### ALK

Anaplastic lymphoma kinase (ALK) alterations are found in approximately 3-5% of all lung adenocarcinomas and are mostly found in younger patients with light- or never-smoking history; approximately 30% of patients will have CNS metastases at the time of diagnosis (113, 114). There are multiple ALK-TKI agents approved in the US for advanced or metastatic *ALK*-positive NSCLC, including first-generation crizotinib, second-generation ceritinib, alectinib, ensartinib, and brigatinib, and third-generation lorlatinib. The phase III ALEX trial demonstrated superiority of alectinib compared to crizotinib in the first-line setting (115, 116). Lorlatinib, a third-generation ALK and ROS-1 inhibitor has shown significant improvements in five-year PFS (60% vs 8%) and time to intracranial progression (not reached vs 16.4 months) compared to crizotinib in the phase III CROWN trial (117). Newer ALK TKIs are being investigated, including neladalkib, a highly selective ALK-inhibitor (118); the phase III ALKAZAR study will compare this agent to alectinib in the front-line setting (NCT06765109). In early-stage disease, the global phase III ALINA trial compared alectinib to platinum-based chemotherapy in the adjuvant setting in completely resected stage IB-IIIA (AJCC 7^th^ edition) *ALK*-positive NSCLC and demonstrated improved disease-free survival (119). Clinical trials exploring combination therapies with anti-VEGF agents, combination TKIs, concurrent chemotherapy, and other novel agents are ongoing.

### ROS1

Proto-oncogene tyrosine-protein kinase-1 (*ROS1*) gene fusions are found in approximately 1-2% of patients with NSCLC and are associated with younger age at diagnosis, light- or never-smoking history, adenocarcinoma histology, and are typically mutually exclusive from other oncogenic driver alterations in *de novo* disease (120). At the time of diagnosis, approximately 85% will have metastatic disease and 20-40% will be found to have brain metastases (121, 122). In advanced or metastatic disease, the first approved agent was the multi-kinase inhibitor, crizotinib, based on the multicenter phase I PROFILE 1001 trial (123, 124). Entrectinib, a multi-kinase inhibitor with improved CNS activity, was approved for use in advanced disease based on a combined analysis of STARTRK-1, STARTRK-2 and ALKA-372–001 clinical trials (125). Repotrectinib, a next-generation ROS1 inhibitor, was shown to have activity in both ROS-TKI-naïve and ROS1-TKI-pretreated patients in the phase I/II TRIDENT-1 clinical trial; in previously untreated patients, the ORR was 79%, the intracranial ORR was 89%, the median DOR was 34.1 months, and the median PFS was 35.7 months (126). Phase III trials are ongoing directly comparing entrectinib and repotrectinib to crizotinib (NCT04503807, NCT06140836). More recently, taletrectinib, a selective ROS1-TKI with CNS activity, has been approved in June 2025 for patients with previously treated or untreated ROS1-NSCLC based on a pooled analysis of the TRUST-I and TRUST-II clinical trials; in treatment-naïve patients, the ORR was 88.8%, the intracranial ORR was 76.5%, the median DOR was 44.2 months, and the median PFS was 45.6 months (127). Ongoing investigations include agents such as zidesamtinib, a CNS-penetrant, ROS1-selective inhibitor is currently being evaluated in the ARROS-1 clinical trial (NCT05118789).

### RET

Rearranged during transfection (*RET*) gene alterations, either by point mutations or gene fusions, are implicated in several malignancies, most commonly thyroid cancers and NSCLC. *RET* gene fusions are found in approximately 1-2% of NSCLC and are associated with younger age at diagnosis, light- or never-smoking history, adenocarcinoma histology, and typically are mutually exclusive from other oncogenic driver alterations in *de novo* disease (128, 129). At the time of diagnosis, a majority of patients will have metastatic disease and approximately 20% will be found to have brain metastases (128, 130). Patients with *RET*-rearranged NSCLC typically have lower PD-L1 expression and tumor mutation burden (130). The most common fusion partner is *KIF5B* followed by *CCDC6*, with no current evidence for a difference in prognosis across treatment types among the various fusion partners (130).

Historically, chemotherapy had been utilized in the front-line setting for advanced disease, with variable efficacy (131, 132). Multi-kinase inhibitors, including cabozantinib, vandetanib, and lenvatinib have only demonstrated modest clinical benefit (132–136). The selective RET-inhibitors, selpercatinib and pralsetinib, are approved as front-line agents in *RET-*fusion NSCLC. The phase III LIBRETTO-431 trial compared selpercatinib to platinum-based chemotherapy with or without immunotherapy and demonstrated an improved median PFS of 24.8 months vs 11.2 months (HR 0.46, p<0.001) (137). Pralsetinib was studied in the phase I/II single-arm, multicenter ARROW trial with prior platinum-based chemotherapy and treatment-naïve cohorts; in the final update, ORR in patients with prior platinum-based chemotherapy was 63.1% vs 78.3% in treatment-naïve patients, with a median DOR of 31.8 months and 13.4 months, respectively (138). The phase III AcceleRET-Lung trial (NCT04222972) is an ongoing effort evaluating pralsetinib vs platinum-based chemotherapy with or without pembrolizumab in the front-line setting.

### BRAF V600E

B-Raf proto-oncogene (*BRAF*) activating mutations are found in up to 4.5% of NSCLC (139). While there are three classes of *BRAF* mutations, currently approved BRAF inhibitors in NSCLC effectively inhibit only class I mutations occurring on codon 600, with the most prevalent being *BRAF V600E*, leading to a RAS-independent constitutively active protein (140). Epidemiological studies of age, sex and smoking history are varied in BRAF-mutated NSCLC with prognostic implications being largely unknown (140). While retrospective studies have reported poorer outcomes with platinum-based chemotherapy in patients *BRAF V600E* mutated disease compared to wild-type, combination chemoimmunotherapy has been shown to be effective in this population, with recent retrospective studies showing similar or improved overall survival in the front-line setting with chemo-immunotherapy compared to front-line BRAF/MEK-inhibitor therapy among specific subpopulations (140–143); therefore, front-line treatment choice must be made on disease factors and with informed decision-making with each patient.

Currently approved front-line combination therapy in BRAF *V600E*-mutated advanced NSCLC includes dabrafenib-trametinib and encorafenib-binimetinib. In a phase II, multi-center, open-label, non-randomized trial, dabrafenib-trametinib demonstrated robust clinical activity in pretreated and treatment naïve patient cohorts (144). In the single-arm, open-label, phase II PHAROS trial, the combination of encorafenib-binimetinib was evaluated among pretreated and treatment naïve cohorts for patients with *BRAF V600E*-mutated NSCLC with high rates of activity in the treatment naïve cohort; specifically, the combination demonstrated an ORR of 75%, a median DOR of 40.0 months, a median PFS of 30.4 months, and a median OS of 47.5 months in the treatment-naïve cohort after a median follow-up time of 52.3 months (145, 146). A similar phase II clinical trial, ENCO-BRAF (NCT04526782) is ongoing, along with IO-targeted therapy combinations and novel BRAF inhibitors.

### MET

Alterations in MNNG-HOS transforming gene (*METI* in NSCLC can include amplifications (increased gene copies, 1-6%), *MET* exon 14 skipping mutations (METex14, 2-4%), overexpression (increased cellular protein expression, 20-25%) and MET fusion (0.2-0.3%) (147). *MET* amplification can be detected via FISH or NGS, with the former being a standard method in clinical practice and trials (148); while the definition of *MET* amplification varies across studies, a *MET-centromere 7 (CEP7)* copy-number ratio of at least 1.8 via FISH is required to be defined as amplified (148). MET overexpression can be determined by IHC, with the definition of overexpression remaining controversial due to the limited standardized cut-off scores, semi-subjective nature of IHC scoring, and values used in clinical trials (148, 149). Lastly, *MET* mutations and fusions can be detected by NGS.

*MET* amplification has been implicated in EGFR-TKI resistance and an increased copy number confers a worse prognosis in NSCLC (150–152); additionally, *MET* amplification has been implicated in bypass mechanisms of resistance to *KRAS G12C* inhibitors and in a minority of patients treated with selpercatinib in *RET-*fusion positive advanced NSCLC (153, 154). The *MET* exon 14 (METex14) skipping mutation is a well-recognized oncogenic driver in NSCLC and independently portends a poor prognosis and OS in NSCLC (155); patients with this alteration tend to be older females and are less likely to have a significant smoking history (156). Despite a significant number of METex14-mutated NSCLC expressing high PD-L1 TPS, the clinical utility of IO remains unclear given variable response rates without targeted therapy and unimproved efficacy or tolerability of IO when combined with targeted therapy (148, 157). Lastly, while the prognostic significance of MET overexpression had been previously debated, the approval of the MET-directed antibody–drug conjugate telisotuzumab-vedotin (Teliso-V) has established MET overexpression as a predictive biomarker (148).

Currently approved agents in the U.S. for METex14-mutated advanced NSCLC include capmatinib and tepotinib. Capmatinib was evaluated in the open-label, non-randomized, multicenter phase II GEOMETRY mono-1 trial and demonstrated clinical benefit amongst both treatment-naïve and pretreated patient cohorts (158). Tepotinib was evaluated in the open-label, non-randomized, multicenter phase II VISION trial and similarly demonstrated benefit among treatment-naïve and pretreated patients (159). Of note, subgroup analyses in both GEOMETRY-mono-1 and VISION trials for patients with *MET* amplification, defined as ≥10 MET gene copy numbers and ≥2.5, respectively, showed clinical efficacy with the use of these agents (160, 161).

Given the increased frequency of MET overexpression in NSCLC compared to MET-mutated disease and limited targeted therapy options in this patient population, MET-directed antibodies have become an attractive approach for therapeutic intervention. Telisotuzumab-vedotin (Teliso-V), an ADC linking a monoclonal antibody against MET to a microtubule inhibitor monomethyl auristatin E (MMAE) payload, has been recently approved for patients with previously treated, EGFR wild-type, MET-high overexpressed, advanced nonsquamous NSCLC based on results from the phase II LUMINOSITY trial (28). In this study, MET-high expression was defined as IHC membrane staining of 3+ intensity in ≥ 50% of tumor cells. While the trial included a separate cohort of patients with MET-intermediate expression (IHC 3+ staining on ≥ 25 to < 50% of cells), a greater ORR was seen in the MET-high cohort; notably, other clinical outcomes, including PFS and OS, were comparable among MET-high and MET-intermediate groups (28). Additional clinical trials exploring the application of Teliso-V are ongoing (NCT06568939, NCT04928846), with its use as combination therapy in patients with MET-overexpression and concurrent *EGFR-*mutated NSCLC previously explored in early phase studies (162, 163).

### KRAS G12C

Kirsten rat sarcoma virus (*KRAS*) gene mutations occur in approximately 25-30% of NSCLC, with the most common being *KRAS G12C*, followed by *G12V* and *G12D* (164). Several tumor suppressor genes have been found and occur as co-mutations with *KRAS*, including *STK11* and *KEAP1* which are associated with immunotherapy resistance and poor prognosis, as discussed previously, and *TP53*, which has been linked to increased immunotherapy sensitivity (17, 18, 165). Currently, combination chemoimmunotherapy remains standard of care as front-line setting in advanced or metastatic NSCLC with *KRAS* mutations. For patients with *KRAS G12C*-mutated advanced or metastatic NSCLC, sotorasib and adagrasib are the only approved targeted agents for use in the second-line or later setting. Sotorasib was approved based on data from the CodeBreaK-100 multicenter, open-label, single-arm, phase I/II clinical trial of patients who previously had received platinum-based chemotherapy with or without IO (166, 167). Sotorasib was subsequently compared to standard second-line docetaxel in the phase III CodeBreaK-200 clinical trial and confirmed an improved ORR and PFS but failed to demonstrate OS benefit (168, 169). Adagrasib was similarly evaluated in previously treated patients in the phase I/II KRYSTAL-1 clinical trial (170); when compared to docetaxel, adagrasib showed an improved ORR and median PFS in the phase III KRYSTAL-12 clinical trial (171).

The integration of *KRAS*-targeting agents in the front-line setting and the development of additional *KRA*S-targeted agents are currently being explored. Olomorasib is a next-generation *KRAS G12C*-targeting agent being explored in the front-line setting as combination therapy with chemoimmunotherapy in the LOXO-RAS-20001 (NCT04956640) and SUNRAY-01 (NCT06119581) clinical trials. Divarasib, another next-generation *KRAS G12C* inhibitor, is being studied in an ongoing, open-label, multicenter phase I clinical trial (NCT04449874) as monotherapy in the second- or later-line setting with promising activity after one year of follow-up (172). Divarasib is also being investigated in the front-line setting for previously untreated patients with *KRAS G12C*-positive NSCLC in the phase Ib/II KRAScendo 170 trial (NCT05789082).

### HER2/ERBB2

Human epidermal growth factor receptor 2 (*HER2*) alterations can include mutations (1-4%), amplifications (2-20%), and overexpression (2-35%) in NSCLC (173, 174). *HER2* mutations are more common in adenocarcinoma histology and typically occur in younger females with light- to never-smoking history, while *HER2* amplifications and overexpression are more commonly seen in male smokers across other histologic subtypes (175–183). *HER2* alterations are associated with worse survival and approximately 19-30% of patients will have brain metastases at diagnosis (181, 184). In the front-line setting for patients with advanced or metastatic *HER2*-altered NSCLC, standard of care remains combination platinum-based chemotherapy with IO. In the second-line setting, HER2-targeted therapy is approved for patients with HER2-mutated or HER2-overexpressing metastatic NSCLC. Currently, the approved agents include the ADC trastuzumab-deruxtecan (T-DXd) for HER2-mutated and overexpressing disease and the TKIs zongertinib and sevabertinib for HER2-mutated disease.

T-DXd is a HER2-targeting ADC, linking trastuzumab to a topoisomerase I inhibitor payload, and was shown to demonstrate clinical benefit in the phase II DESTINY-Lung01 trial in HER2-mutated NSCLC (185). In the phase II DESTINY-Lung02 trial, T-DXd was evaluated at 5.4mg/kg and 6.4mg/kg dosing in patients with HER2-mutated NSCLC with results demonstrating similar ORR between dosing groups but with an improved median OS with the lower dose; additionally, a reduced rate of grade ≥ 3 pneumonitis (14.9% in T-DXd 5.4mg/kg arm, 32% in T-DXd 6.4mg/kg arm) and other adverse events (39% vs 60%) were seen in patients who received the 5.4mg/kg dosing (186, 187). Given these data, T-DXd is approved in the second-line setting at 5.4mg/kg dosing in patients with HER2-mutated NSCLC. The ongoing phase III DESTINY-Lung04 clinical trial will address the efficacy of T-DXd against standard of care platinum-doublet chemotherapy with IO in the front-line setting for patients with HER2-mutated NSCLC (NCT05048797). Lastly, T-DXd has tumor-agnostic approval for use in solid tumors with HER2 overexpression (IHC 3+) in advanced or metastatic NSCLC (26, 27).

Zongertinib is an irreversible HER2-specific TKI which is approved in second-line HER2-mutated advanced or metastatic NSCLC based on the Beamion LUNG-1 multi-cohort phase Ia/Ib trial; robust clinical activity was seen in the cohort of treatment-naïve patients with *HER2* mutation in the tyrosine kinase domain (TKD) (Cohort 1) (188, 189). The phase III Beamion LUNG-2 trial (NCT06151574) evaluating single-agent zongertinib compared to current standard of care platinum-doublet chemotherapy with IO in the front-line setting is ongoing for previously untreated HER2-mutated (in TKD) NSCLC. Additional studies investigating novel HER2-targeting TKIs, ADCs, and combination strategies are ongoing, with sevabertinib showing promising results in the phase I-II SOHO-01 clinical trial (NCT05099172) for *HER2*-mutated disease, leading to its accelerated FDA approval in November 2025 (190); this agent is also being studied as front-line therapy in the ongoing phase III SOHO-02 trial (NCT06452277).

### NTRK fusions

Neurotrophic tropomyosin receptor kinase 1/2/3 (*NTRK 1/2/3*) gene fusions are found in approximately 1% of solid tumors with an estimated incidence of 0.1-0.2% in NSCLC (191, 192). NTRK fusions are more commonly identified in younger individuals with minimal to no smoking history and typically do not overlap with other oncogenic drivers (192). While there are multiple methods for identifying NTRK fusions, RNS-based NGS testing may improve detection over DNA-based sequencing and is the preferred modality (193). Larotrectinib, entrectinib, and repotrectinib are currently FDA approved TRK inhibitors in patients with NTRK-fusion solid tumors based on multiple single-arm clinical trials (125, 194–196); notably, NCCN guidelines lists these agents as first- or second-line options.

### NRG1 fusions

Neurogrelin 1 (*NRG1*) fusions are found in approximately 0.2% of all solid tumors; the estimated incidence in NSCLC is 0.1% (197). Biologically, NRG1 acts as a ligand to ErbB3 (HER3) via an EGF-like domain, most commonly leading to dimerization with HER2 and subsequent downstream signaling of the MAPK and PI3K/AKT pathways (198). Historically, NRG1 fusion-positive NSCLC respond poorly to chemotherapy and immunotherapy (199). NRG1 fusions have recently emerged as a predictive biomarker for targeted therapy, highlighted by the approval of zenocutuzumab, a bispecific antibody against HER2 and HER3 that effectively blocks the NRG1 binding site, which has shown activity in previously treated NSCLC and in pancreatic adenocarcinoma based on the multicenter, open-label, multi-cohort phase II eNRGY study (200, 201). Additional studies targeting NRG1 and/or HER3 are ongoing.

## Discussion

In this review, we have summarized the molecular methods for identifying oncogenic driver alterations in NSCLC with a subsequent highlight of the current biomarkers predictive of response to immunotherapy and targeted therapies. In this current era of targeted treatment options, it is imperative to conduct a thorough evaluation for oncogenic driver alterations, aside from comprehensive NGS testing, given recent drug approvals based on IHC overexpression. Obtaining adequate tumor sample specimens to conduct multimodal molecular testing can be challenging, costly, and time-consuming, which may lead to delays in care; reflexive IHC and NGS testing at the time of tissue-biopsy diagnosis should be considered for at least all patients with advanced or metastatic nonsquamous NSCLC.

Most patients with advanced or metastatic NSCLC, particularly those with squamous histology, receive immunotherapy as part of systemic treatment, in part because AGAs with approved targeted therapies are infrequently identified in this population. For this reason, the development of validated and standardized biomarkers predictive of response to immunotherapy is critical. Resistance to immunotherapy can occur via a variety of mechanisms, including tumor-intrinsic properties (oncogenic driver mutations, co-mutations, impaired antigen presentation), tumor-extrinsic properties (lack of T-cell priming, immunosuppressive TME), changes in adaptive immunity (T-cell exhaustion, loss of tumor neoantigens, upregulation of alternative immune checkpoints), epigenetic modifications, and clonal evolution (202, 203). Novel immunotherapy agents are in development to attempt to overcome resistance pathways and increase efficacy when compared to PD-1/PD-L1 inhibitors. Ivonescimab, a bispecific anti-PD-1 and anti-VEGF antibody was shown to improve PFS compared to pembrolizumab in patients with advanced or metastatic PD-L1-positive NSCLC in the HARMONi-2 clinical trial (204); similarly, the phase III HARMONi-6 trial comparing ivonescimab versus tislelizumab, an anti-PD-1 antibody, in combination with chemotherapy for untreated advanced squamous NSCLC demonstrated improved PFS with the ivonescimab combination (205). The HARMONi-3 clinical trial aims to address the use of ivonescimab versus pembrolizumab in combination with chemotherapy for first-line treatment of metastatic NSCLC and is ongoing (NCT05899608).

Owing to the rarity with which several of the AGAs exist within NSCLC, clinical trials are often limited to the phase II setting with sparse comparative analyses. With many targeted therapies approved in the front-line setting across the varying oncogenic driver alterations identified (e.g. capmatinib and tepotinib in METex14 NSCLC; selpercatinib and pralsetinib in *RET*-fusion NSCLC), it is often felt that these agents have similar efficacy, and thus it is not considered beneficial to alternate to a similar agent upon inevitable disease progression. Analogously, the sequencing of therapies after disease progression can be challenging with the most appropriate salvage therapy at times unclear; for example, with amivantamab and lazertinib combination therapy approved in the first-line setting for sensitizing *EGFR*-mutated NSCLC (MARIPOSA regimen), the role of osimertinib with or without platinum-chemotherapy (FLAURA2 regimen) at progression is unknown. More studies are warranted to help physicians delineate appropriate sequencing of therapies in this setting.

For all patients with AGA NSCLC, disease progression is foreseeable largely due to disease refractoriness to therapy. This can be due to on-target and off-target resistance mechanisms. For example, *EGFR* exon 20 *T790M* mutations, *EGFR* exon 20 *C797S* mutations, and secondary *KRAS* or *RET* mutations can lead to reduced drug efficacy (128, 150, 151, 165, 206). In some circumstances, on-target mutations can lead to the need to switch to an alternative TKI with known coverage, such as the use of osimertinib in EGFR exon 20 *T790M* mutated disease and repotrectinib or taletrectinib in *ROS1 G2032R* mutated disease (120, 150). In other cases, on-target mutations can lead to the need for a complete change in therapy, such as *ROS1 L2086F*-mutated disease which is known to be resistant to all type 1 ROS1 TKIs (120, 150); to date, only cabozantinib, gilteritinib, merestinib, and ceritinib have published pre-clinical activity for *ROS1 L2086R-*mutated disease, with cabozantinib showing clinical efficacy in case reports after use of crizotinib and off-label lorlatinib (207–210).

Off-target mechanisms of resistance can develop via cellular bypass mechanisms or concurrent off-target mutations; for example, *EGFR* amplification*, MET* amplification or overexpression, or *HER2* amplification or overexpression are linked to disease resistance in *EGFR-*mutated disease, and off-target concurrent mutations, including *TP53*, *PTEN*, *PI3KA*, and *KRAS* mutations are linked to resistance in NSCLC across multiple molecular alterations (151). The bispecific antibody amivantamab represents one example of a therapeutic agent designed to overcome resistance mechanisms. Accordingly, next-generation TKIs and combination approaches are currently under development and clinical investigation. Lastly, in rare circumstances, resistance can emerge due to histologic transformation, such as *EGFR*-mutated NSCLC transforming into small-cell lung cancer (211).

In addition to newer generation TKIs and combination strategies for patients with targetable alterations, novel TKIs and emerging targetable biomarkers are an ongoing area of research. One such area includes the study of pan-*RAS* inhibitors, allowing the bypass of resistance mechanisms and the ability of their use in *KRAS* non-*G12C* disease, with several investigational agents being studied in patients with NSCLC and other solid tumors (212). Of the emerging biomarkers predictive of response to targeted therapy, S-methyl-5’-thioadenosine phosphorylase (*MTAP*)-deficiency has shown promising development in NSCLC; while *MTAP* loss has served as a negative predictor of benefit to IO-therapy, cooperative protein arginine N-methyltransferase 5 (PRMT5) inhibitors are being studied in *MTAP*-loss solid tumors (213). Similarly, fibroblast growth factor receptor (*FGFR*) fusions (*FGFR1-3*) are frequently identified in NSCLC; while there are no currently *FGFR*-targeted therapies approved in NSCLC, pemigatinib and futibatinib are approved in advanced cholangiocarcinoma with *FGFR2* fusions while erdafitinib is approved in *FGFR2/3*-altered metastatic urothelial carcinoma (214). The phase I clinical trial evaluating LOXO-435 with *FGFR3*-altered advanced solid tumors, including NSCLC, is underway (NCT05614739). Lastly, studies evaluating novel TROP-2 inhibitors, in addition to datopotamab deruxtecan (Dato-DXd), are ongoing (214).

In conclusion, the identification of predictive biomarkers has fundamentally transformed the therapeutic landscape of NSCLC by enabling the use of targeted therapies in both early-stage and metastatic disease. Establishing streamlined approaches to ensure comprehensive molecular testing remains essential to optimize patient care. Active clinical investigations exploring combination regimens, novel immunotherapy agents, next-generation and novel TKIs, as well as the discovery of additional actionable biomarkers, are expected to further transform and advance this rapidly evolving field.
